# Lymphocyte-to-Monocyte Ratio (LMR) During Induction Is a Better Predictor Than Preoperative LMR in Patients Receiving Intravesical Bacillus Calmette -Guerin for Non-Muscle-Invasive Bladder Cancer

**DOI:** 10.3389/fonc.2022.937638

**Published:** 2022-07-12

**Authors:** Deng-xiong Li, Xiao-ming Wang, De-chao Feng, Fa-cai Zhang, Rui-cheng Wu, Xu Shi, Kai Chen, Yunjin Bai, Ping Han

**Affiliations:** Department of Urology, Institute of Urology, West China Hospital, Sichuan University, Chengdu, China

**Keywords:** non-muscle-invasive bladder cancer, Bacillus Calmette-Guérin, immunotherapeutic, prognosis (carcinoma), LMR

## Abstract

The prognostic value of the lymphocyte-to-monocyte ratio during induction (ILMR) remains unclear in non-muscle-invasive bladder cancer (NMIBC) patients receiving Bacillus Calmette-Guérin (BCG). We aimed to determine and compare the prognostic value of the ILMR, preoperative lymphocyte-to-monocyte ratio (PLMR) and their dynamic changes (PILMR). This study collected the data from NMIBC patients receiving BCG treatment in our institution. The prognostic value of the PLMR, ILMR and PILMR was analyzed by the Kaplan−Meier method and Cox proportional hazard regression models. The concordance index and receiver operating characteristic curve analysis were employed to compare the prognostic value of these three factors. Our study enrolled 197 patients. These patients included 170 male patients, and the mean age was 64.17 years. During the follow-up time, 85 patients experienced recurrence, and 55 patients experienced progression. According to the results of COX multivariable analysis, PLMR (P=0.011) and ILMR (P<0.001) could independently predict the recurrence of NMIBC patients receiving BCG. Meanwhile, ILMR (P=0.001) and PILMR (P=0.036) were also the independent prognostic factors of progression. Compared with PLMR and PILMR, ILMR was associated with better accuracy for NMIBC patients receiving BCG. This study first found that the ILMR could independently predict the prognosis of NMIBC patients receiving BCG. Furthermore, we also identified that ILMR was associated with higher prognostic value than PLMR and PILMR, which might help to select an optimal treatment schedule for patients with NMIBC.

## Introduction

Bladder cancer is the 10th most commonly diagnosed carcinoma worldwide and caused 213,000 deaths in 2020 (1). Non-muscle-invasive bladder cancer (NMIBC) occupies approximately 70% newly diagnosed bladder cancers and easily recurs and progresses after transurethral resection of bladder tumor (TURBT). Fortunately, since Bacillus Calmette-Guérin (BCG) was used clinically, the rates of recurrence and progression have obviously declined in recent decades. Nevertheless, almost 30% of NMIBC patients do not respond to BCG treatment (2). For patients with BCG failure or high-risk patients, various aggressive therapies are employed to improve the prognosis of these patients. For instance, some urologists suggested that patients with BCG failure or high-risk patients might accept early radical cystectomy rather than intravesical therapies (2–4). However, radical cystectomy has considerable morbidity, even in high-volume centers of excellence and regardless of open versus robotic approaches (3, 5, 6). Given this condition, urologists must weigh carefully to determine whether aggressive treatments should be performed. Various methods, including DNA methylation and mutations, protein-based assays, non-coding RNAs and mRNA signatures, were used for risk stratification in NMIBC patients (7). However, these biomarkers are not easy to perform and need further improvement. Thus, urologists are continually devoted to finding a readily available and powerful prognostic factor to screen out patients who have no response to BCG and should accept early aggressive treatment.

It is widely reported that the preoperative lymphocyte-to-monocyte ratio can independently predict the prognosis of many malignant tumors, such as gastric, ovarian, kidney and prostate cancers (8). Recent evidence suggests that preoperative lymphocyte-to-monocyte ratio (PLMR) can be an independent prognostic factor for patients receiving BCG and is associated with pathological features (9–11). Trained immunity is an important mechanism mediating BCG immunotherapy (12). Cytokines, including IL-1β, TNF, IL-10 and GM-CSF, are significantly increased after the first BCG instillation (13, 14) and can also predict the recurrence of patients receiving BCG (14). Monocyte cells, a kind of innate immune cell, can be trained by BCG through producing cytokines, such as TNF and IL-6 (15, 16). Arts et al. (15) reported that the number of monocyte cells was significantly increased at day 6 after culturing with BCG. Similarly, Bisiaux et al. (13) observed an infiltration of monocytes at 4 hours after the first BCG induction instillation in NMIBC patients. Thus, we reasonably assumed that the lymphocyte-to-monocyte ratio during induction (ILMR) might be a powerful prognostic factor for NMIBC patients receiving BCG. However, no study has reported the prognostic value of the ILMR in patients who received BCG.

To clarify the prognostic value of the ILMR, we collected data from NMIBC patients receiving BCG treatment in our institution after approval by the ethics committee. The first purpose was to identify the prognostic value of the PLMR, ILMR and their dynamic change (PILMR). The second purpose was to compare the prognostic value of these three factors.

## Materials and Methods

### Patient Selection and Data Collection

We collected the data of NMIBC patients receiving BCG in our hospital from 2014 to 2020 after approval by our institutional ethics committee (approval number: 20201045). Patients included in our study should have LMR data before surgery and during BCG induction. The exclusion criteria for selecting the patients were as follows: patients with diseases that could affect the results of blood counts (such as prostatitis, cystitis, urinary tract infection, yeast infections, endometriosis and systemic inflammatory disease), those with missing data, other sites of carcinoma, and those who received other intravesical chemotherapy (except immediate single instillation after TURBT).

Experienced pathologists carefully evaluated all specimens included in this study. Pathological information, including tumor stage, World Health Organization (WHO) grade and carcinoma *in situ* (CIS), was reviewed referring to the 2016 WHO bladder cancer classification (17) and the 2016 American Joint Committee on Cancer (18). Of these, NMIBC would be considered histological variant (HV) NMIBC if any HV appeared in the specimens. Moreover, demographic and clinical outcomes were also determined in this study.

### Patient Management and Follow-up

All patients in this study received intravesical BCG therapy. Specifically, induction BCG instillations were performed once a week for 6 weeks. After BCG induction, at least one year of maintenance instillations would be given if the patient’s condition permits (2). Patients accepted cystoscopy and urinary cytology every 3 months for the first year, every 6 months 2 to 5 years after TURBT, and then annually. Moreover, imaging and laboratory examination were performed if patients were in need. Recurrence-free survival (RFS) was defined as the time from the date of surgery to local or distant recurrence. Progression-free survival (PFS) was defined as an increase in the stage to MIBC and/or metastasis.

### Statistical Analysis

PLMR and ILMR were calculated by dividing the absolute lymphocyte count by the absolute monocyte count, while PILMR was generated by dividing PLMR by ILMR. In detail, the PLMR should be determined two weeks before surgery, while the ILMR was generated by blood counts measured during BCG induction. Categorical and continuous variables between groups were analyzed using Chi-square and Student’s t-tests, respectively. Fisher’s exact test was applied to estimate categorical variables when one or more of the cell counts in a 2×2 table were less than 5. The best cut-off values of PLMR and ILMR were generated by the receiver operating characteristic curve (ROC) and Youden index. The prognosis, including RFS and PFS, was validated by the Kaplan−Meier method, and comparisons between groups were performed by log-rank tests. Univariable and multivariable Cox proportional hazard regression models were employed to evaluate the association of PLMR, ILMR and PILMR with RFS and PFS. Referring to the existing model, univariable Cox proportional hazard regression analysis assessed the prognostic value of many factors, such as age, gender, tumor number, T stage, concomitant CIS, and WHO tumor grade (19). Moreover, many clinical factors, including smoking history, BMI, gross hematuria, hypertension, diabetes, tumor size and HV, were also evaluated by univariable Cox proportional hazard regression analysis. Then, according to the results of univariable Cox proportional hazard regression analysis, the factors with P value <0.05 were included in multivariable Cox proportional hazard regression model. To compare the prognostic value of the PLMR, ILMR and PILMR, we constructed RFS and PFS Cox proportional hazard regression models based on same clinical factors and different LMR. These models that separately evaluated different LMR could avoid the influence of possible multicollinearity. After building these models, the concordance index (C-index) and multiparameter ROC analysis were used to validate the accuracy of different multivariable Cox proportional hazard regression models. A P<0.05 was considered significant for all analyses, which were performed using R version 3.6.3 and relative packages.

## Results

### Demographic and Clinicopathologic Characteristics

Our study finally enrolled 197 patients. The mean ± standard deviation (SD) follow-up time was 30.18 ± 15.67 months. For PLMR and ILMR, the optimal cut-off values were 3.5 and 3.6, respectively. Then, referring to the cut-off value, all patients were divided into high and low groups. In the PILMR, patients with a PILMR >1 were regarded as the high PILMR group, and the others were regarded as the low PILMR group. A total of 21/197 (10.6%) patients exhibited side effects, with cystitis being the most common. To compare the difference between the high and low groups, **Table 1** showed that patients in the high PLMR group were older than those in low PLMR group. High ILMR was correlated with high body mass index (BMI), low mean age and large tumors. High PILMR was significantly associated with high BMI and high WHO grade. The remaining detailed information was provided in **Table 1**.

**Table 1:** Demographic and clinicopathologic characteristics.

	Total	PLMR			ILMR			BILMR		
	n = 197	Low (133)	High (64)	P value	Low (94)	High (103)	P value	Low (118)	High (79)	P value
Sex				0.148			0.026			0.575
Female	27 (13.7%)	22 (11.2%)	5 (2.5%)		7 (3.6%)	20 (10.2%)		18 (9.1%)	9 (4.6%)	
Male	170 (86.3%)	111 (56.3%)	59 (29.9%)		87 (44.2%)	83 (42.1%)		100 (50.8%)	70 (35.5%)	
Age	64.17	62.44	67.77	0.001	66.46	62.08	0.005	64.13	64.23	0.950
	±11.05	± 11.2	± 9.97		± 10.9	± 10.87		± 11.04	± 11.2	
BMI	23.65	23.63	23.68	0.925	22.7	24.51	< 0.001	23.15	24.39	0.005
	±3.04	± 3.01	± 3.14		± 2.87	± 2.95		± 2.83	± 3.22	
Smoker				0.092			0.682			0.162
No	140 (71.1%)	89 (45.2%)	51 (25.9%)		65 (33%)	75 (38.1%)		79 (40.1%)	61 (31%)	
Yes	57 (28.9%)	44 (22.3%)	13 (6.6%)		29 (14.7%)	28 (14.2%)		39 (19.8%)	18 (9.1%)	
Gross hematuria				0.358			0.390			0.294
No	46 (23.4%)	28 (14.2%)	18 (9.1%)		25 (12.7%)	21 (10.7%)		24 (12.2%)	22 (11.2%)	
Yes	151 (76.6%)	105 (53.3%)	46 (23.4%)		69 (35%)	82 (41.6%)		94 (47.7%)	57 (28.9%)	
Hypertension				0.182			0.682			1.000
No	140 (71.1%)	99 (50.3%)	41 (20.8%)		65 (33%)	75 (38.1%)		84 (42.6%)	56 (28.4%)	
Yes	57 (28.9%)	34 (17.3%)	23 (11.7%)		29 (14.7%)	28 (14.2%)		34 (17.3%)	23 (11.7%)	
Diabetes				0.529			0.854			1.000
No	172 (87.3%)	118 (59.9%)	54 (27.4%)		83 (42.1%)	89 (45.2%)		103 (52.3%)	69 (35%)	
Yes	25 (12.7%)	15 (7.6%)	10 (5.1%)		11 (5.6%)	14 (7.1%)		15 (7.6%)	10 (5.1%)	
Tumor size^a^				0.942			0.045			0.321
<=3cm	110 (55.8%)	75 (38.1%)	35 (17.8%)		45 (22.8%)	65 (33%)		62 (31.5%)	48 (24.4%)	
>3cm	87 (44.2%)	58 (29.4%)	29 (14.7%)		49 (24.9%)	38 (19.3%)		56 (28.4%)	31 (15.7%)	
Tumor number				0.632			0.466			0.240
Single	86 (43.7%)	56 (28.4%)	30 (15.2%)		38 (19.3%)	48 (24.4%)		47 (23.9%)	39 (19.8%)	
Multiple	111 (56.3%)	77 (39.1%)	34 (17.3%)		56 (28.4%)	55 (27.9%)		71 (36%)	40 (20.3%)	
WHO grade				0.580			0.383			0.037
Low	55 (27.9%)	35 (17.8%)	20 (10.2%)		23 (11.7%)	32 (16.2%)		26 (13.2%)	29 (14.7%)	
High	142 (72.1%)	98 (49.7%)	44 (22.3%)		71 (36%)	71 (36%)		92 (46.7%)	50 (25.4%)	
CIS				0.569			0.574			0.868
No	182 (92.4%)	25 (12.7%)	15 (7.6%)		17 (8.6%)	23 (11.7%)		23 (11.7%)	17 (8.6%)	
Yes	15 (7.6%)	108 (54.8%)	49 (24.9%)		77 (39.1%)	80 (40.6%)		95 (48.2%)	62 (31.5%)	
T stage				0.570			1.000			1.000
Ta	40 (20.3%)	124 (62.9%)	58 (29.4%)		87 (44.2%)	95 (48.2%)		109 (55.3%)	73 (37.1%)	
T1	157 (79.7%)	9 (4.6%)	6 (3%)		7 (3.6%)	8 (4.1%)		9 (4.6%)	6 (3%)	
Histology				0.981			0.645			0.975
No HV	171 (86.8%)	116 (58.9%)	55 (27.9%)		80 (40.6%)	91 (46.2%)		103 (52.3%)	68 (34.5%)	
HV	26 (13.2%)	17 (8.6%)	9 (4.6%)		14 (7.1%)	12 (6.1%)		15 (7.6%)	11 (5.6%)	
Recurrence				0.006			0.002			0.448
No	112 (56.9%)	85 (43.1%)	27 (13.7%)		42 (21.3%)	70 (35.5%)		64 (32.5%)	48 (24.4%)	
Yes	85 (43.1%)	48 (24.4%)	37 (18.8%)		52 (26.4%)	33 (16.8%)		54 (27.4%)	31 (15.7%)	
Progression				0.056			0.009			0.408
No	142 (72.1%)	102 (51.8%)	40 (20.3%)		59 (29.9%)	83 (42.1%)		82 (41.6%)	60 (30.5%)	
Yes	55 (27.9%)	31 (15.7%)	24 (12.2%)		35 (17.8%)	20 (10.2%)		36 (18.3%)	19 (9.6%)	

a : For multiple tumors, the diameter of the largest tumor was regarded as tumor size. BMI, Body mass index; WHO, World Health Organization; CIS, Carcinoma in situ; HV, Histological variant; PLMR, Preoperative lymphocyte-to-monocyte ratio; ILMP, Lymphocyte-to-monocyte ratio during induction; PILMR, Preoperative dividing by induction lymphocyte -to- monocyte ratio; n, number.

### The Relationship Between RFS and PLMR, ILMR and PILMR

**Figure 1:**
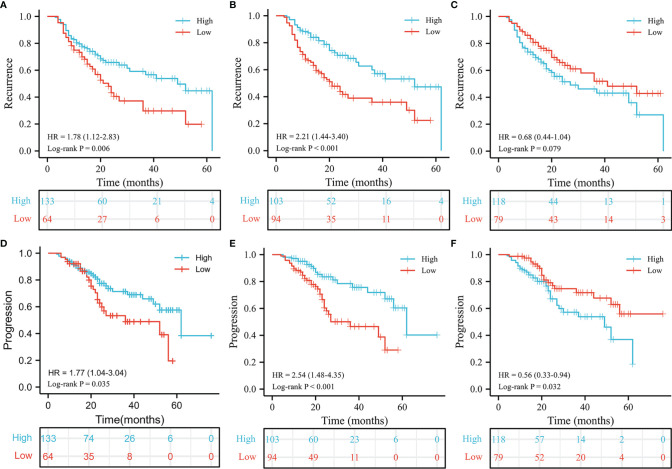
Kaplan-Meier estimates for recurrence and progression. The recurrence results of PLMR **(A)**, ILMR **(B)** and PILMR **(C)**. The progression results of PLMR **(D)**, ILMR **(E)** and PILMR **(F)**.

**Figure 2:**
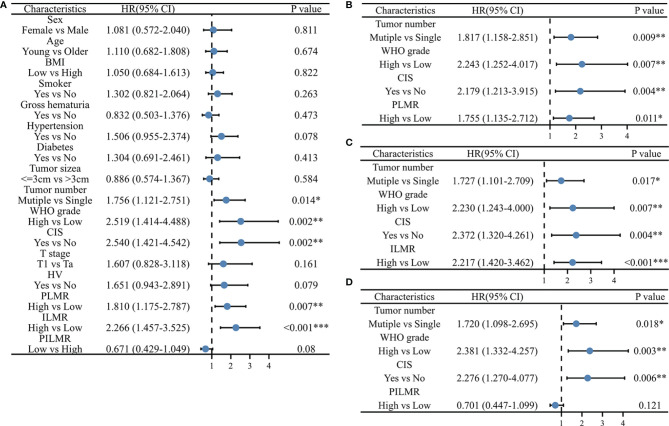
Univariate and multivariate Cox Proportional Hazard model for recurrence: **(A)** univariate results, **(B)** multivariate results with PLMR, **(C)** multivariate results with ILMR, **(D)** multivariate results with PILMR. PLMR, Preoperative neutrophil-to-lymphocyte ratio; ILMP, Lymphocyte-to-monocyte ratio during induction; PILMR, Preoperative dividing by induction lymphocyte-to-monocyte ratio; *: P < 0.05; **:P < 0.01; ***:P < 0.001.

There were 85/197 (43.1%) patients who were diagnosed with recurrence during follow-up. The results of Chi-square analysis showed that patients with a low PLMR were associated with a significantly higher recurrence rate (**Table 1**, P=0.006). The correlation between RFS and PLMR was also identified by the results of log-rank tests (**Figure 1A**, P=0.006) and univariable analysis (**Figure 2A**, P=0.007). Moreover, multivariable analysis demonstrated that the PLMR was an independent prognostic factor for NMIBC patients receiving BCG (**Figure 2B**, P=0.011).

For ILMR, patients with low ILMR were positively related to recurrence rate according to the results of Chi-square analysis (**Table 1**, P=0.002). Similarly, the relationship between RFS and ILMR was identified by the results of log-rank tests (**Figure 1B**, P<0.001) and univariable analysis (**Figure 2A**, P<0.001). Further multivariable analysis illustrated that ILMR was an independent prognostic factor of NMIBC patients receiving BCG (**Figure 2C**, P<0.001). There was no significant difference in RFS between the high and low PILMR groups according to the results of the Chi-square analysis (**Table 1**, P=0.448), log-rank tests (**Figure 1C**, P=0.079), univariable analysis (**Figure 2A**, P=0.080) and multivariable analyses (**Figure 2D**, P=0.121).

### The Relationship Between PFS and PLMR, ILMR and PILMR

**Figure 3:**
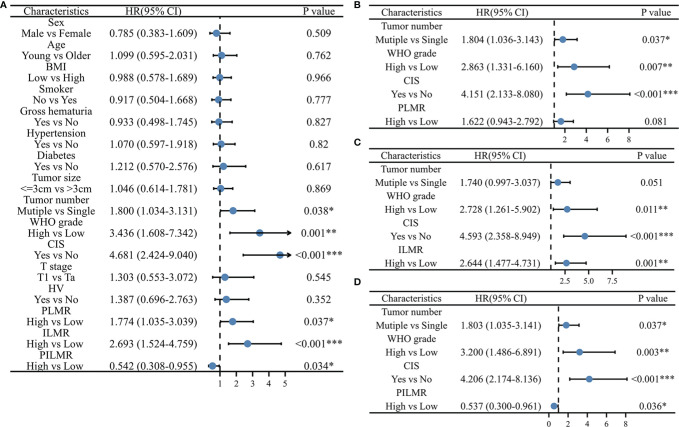
Univariate and multivariate Cox Proportional Hazard model for progression: **(A)** univariate results, **(B)** multivariate results with PLMR, **(C)** multivariate results with ILMR, **(D)** multivariate results with PILMR. PLMR, Preoperative neutrophil-to-lymphocyte ratio; ILMP, Lymphocyte-to-monocyte ratio during induction; PILMR, Preoperative dividing by induction lymphocyte-to-monocyte ratio; *: P < 0.05; **:P < 0.01; ***:P < 0.001.

The total number of progressions was 55/197 (27.9%) at the end of follow-up. Based on the Chi-square analysis, patients with low PLMR were positively associated with the recurrence rate, but no significant difference was found (**Table 1**, P=0.056). It was easier for patients in the low PLMR group to progress according to log-rank tests (**Figure 1D**, P=0.035) and univariable analysis (**Figure 3A**, P=0.037). However, further multivariable analysis indicated that the PLMR could not independently predict the prognosis of NMIBC patients receiving BCG (**Figure 3B**, P=0.081).

Compared with the high ILMR group, the low ILMR group was positively associated with the progression rate according to the Chi-square analysis (**Table 1**, P=0.009), log-rank tests (**Figure 1E**, P<0.001), univariable analysis (**Figure 3A**, P<0.001) and multivariable analysis (**Figure 3C**, P=0.001). Furthermore, ILMR was an independent prognostic factor for NMIBC patients receiving BCG. Although the Chi-square analysis did not show prognostic value (**Table 1**, P=0.408), PILMR was identified as an independent predictor for progression in log-rank tests (**Figure 1F**, P=0.032), univariable analysis (**Figure 3A**, P=0.034) and multivariable analysis (**Figure 3D**, P=0.036).

### Validation of the Accuracy and Reliability of PLMR, ILMR and PILMR

From the data in **Table 2**, the C-index and area under curve (AUC) value of the ILMR were higher than those of PLMR and PILMR in term of recurrence. For progression, the ILMR showed moderate accuracy (**Table 2**, C-index=0.719). Meanwhile, the AUC value of the ILMR was 0.754, which was higher than that of PLMR (**Table 2**, AUC=0.733) and PILMR (**Table 2**, AUC=0.716).

**Table 2:** The accuracy and reliability of PLMR, ILMR and PILMR.

	PLMR		ILMR		IPLMR	
Recurrence						
C-index, 95%CI	0.625	0.593-0.657	0.659	0.627-0.691	0.629	0.597-0.662
AUC, 95%CI	0.814	0.751-0.878	0.826	0.763-0.888	0.798	0.733-0.863
Progression						
C-index, 95%CI	0.678	0.637-0.718	0.719	0.679-0.759	0.703	0.667-0.739
AUC, 95%CI	0.733	0.652-0.813	0.754	0.672-0.836	0.716	0.634-0.797

CI, Confidence level; C-index, concordance index; AUC, area under curve; PLMR, Preoperative lymphocyte-to-monocyte ratio; ILMP, Lymphocyte-to-monocyte ratio during induction; PILMR, Preoperative dividing by induction lymphocyte -to- monocyte ratio.

## Discussion

The prognostic value of the PLMR has widely attracted the attention of doctors. Therefore, urologists also intend to clarify the role of PLMR in BCG treatment to screen out NMIBC patients who may fail to respond to BCG. In the current study, we not only identified that the PLMR had independent prognostic ability, but also illustrated for the first time that the ILMR could independently predict the prognosis of NMIBC patients receiving BCG. Furthermore, compared with the PLMR and PILMR, the ILMR was a better prognostic factor for NMIBC patients receiving BCG.

Tumor-related inflammation has been widely studied and listed as a major phenotype of malignant tumors (20). Meanwhile, inflammatory and immune cells are important parts of the tumor microenvironment and indispensable factors in promoting tumor proliferation, tumor progression, survival and migration (21). Bladder cancer is an inflammation- and immune-related malignant tumor. The inflammatory and immune environments of peripheral blood and tumor are significantly changed during BCG induction (12). Therefore, it is important for urologists to clarify the role of peripheral immune cells in NMIBC.

In this study, in order to exclude the interference of some clinical factors, univariable Cox proportional hazard regression analysis was used to evaluate the prognostic value of multiple factors, such as smoking history, BMI, gross hematuria, hypertension, diabetes, tumor size, tumor number and HV (22). Then, according to the results, the factors with P value <0.05 were included in multivariable Cox proportional hazard regression model. This action ensured the outcomes of this study were reliable. Moreover, we did not warry about the influence of racial disparities due to all patients including in this study were native Chinese (23). Similar to prostate-specific antigen, a widely recognized cut-off value can significantly promote the clinical application of LMR and help select the optimal treatment plan. The optimal cut-off values of the PLMR and ILMR were 3.5 and 3.6 in our study, respectively, which were consistent with most studies. Similarly, Adamkiewicz et al. (10) obtained that the optimal cut-off value of PLMR was 3.25 by analyzing NMIBC patients receiving BCG. This value was in agreement with Yıldız’s (9) finding, which suggested that 3.63 was the best PLMR cut-off value for NMIBC patients receiving BCG. Wang et al. (24) believed that 3.86 was the best cut-off value of PLMR by analyzing stage T1 NMBC patients. Moreover, a study reported that 3.41 was the best cut-off value of PLMR in 1155 high-risk NMIBC patients (25). Mao et al. (26) calculated that the cut-off value of the PLMR was 4.3, which enrolled patients with low-risk NMIBC. These results suggested that the optimal cut-off value was different between low-risk NMIBC and intermediate- or high- risk NMIBC. Despite differences, most cut-off values of PLMR are usually approximately 3.5 in patients with intermediate- or high-risk NMIBC. This phenomenon was beneficial to the clinical practice of PLMR and ILMR.

The prognostic value of the PLMR was first reported in patients undergoing radical cystectomy rather than NMIBC (27). Subsequent studies also demonstrated the prognostic value of the PLMR in muscle invasive bladder cancer (28). An initial objective of the study was to clarify the prognostic value of the PLMR, ILMR and PILMR in NMIBC patients receiving BCG. Recently, several studies have reported that PLMR could predict the prognosis of patients with NMIBC. For instance, Mao et al (26) illustrated that a high PLMR was associated with better RFS and PFS according to the results of log-rank tests. This finding was consistent with Wang et al. (24), who demonstrated that PLMR could independently predict the RFS and PFS of stage T1 NMIBC patients receiving various treatments. Similarly, the prognostic value of the PLMR was also identified in patients with NMIBC receiving BCG by multivariable analysis (10).Furthermore, the PLMR could improve the prognostic value of the Spanish Urological Club for Oncological Treatment (CUETO) (29). This finding was also reported by Yıldız et al. (9). Consistent with these studies, our study found that patients with a high PLMR were associated with better RFS and PFS, again identifying the independent prognostic value of PLMR. No study reported the prognostic value of ILMR and PILMR. In this study, the PILMR failed to be an independent prognostic factor for recurrence. Although PILMR could independently predict the progression of NMIBC patients receiving BCG, its prognostic value was still limited. In current study, we first identified that the ILMR could predict the prognosis of NMIBC patients receiving BCG. Further analysis demonstrated that ILMR had the highest accuracy of all models. ILMR was generated by blood tests measured during BCG induction, so that NMIBC patients could predict the outcome of BCG immunotherapy early and adjust the treatment plan in time. These results supported that ILMR might help urologists select an optimal treatment schedule for patients with NMIBC.

Recently, various biomarkers, including clinical and basic methods, are appearing (30–35). For instance, vesical imaging-reporting and data system (VIRADS) can determine the preoperative stage of TURBT (30, 31). The combination of imaging and ILMR may more accurately screen out patients who have no response to BCG and should accept early aggressive treatment. A few limitations should be mentioned, including those inherent to the retrospective design of the study. The bias of data might be noticed because the data were derived from a hospital information system. Therefore, we used strict exclusion criteria to avoid possible bias. Additionally, although the number of enrolled patients permits us to perform multivariable analysis, prospective and large-scale studies are still required for more representative samples with higher statistical power.

## Conclusion

This study first found ILMR could independently predict the prognosis of NMIBC patients receiving BCG treatment. Furthermore, compared with the PLMR and PILMR, the ILMR had better prognostic value, which might help select an optimal treatment schedule for patients with NMIBC. Of course, prospective and large-scale studies are still required for more representative samples with higher statistical power.

## Data Availability Statement

The raw data supporting the conclusions of this article will be made available by the authors, without undue reservation. Requests to access these datasets should be directed to D-xL, 1010441069@qq.com.

## Ethics Statement

The studies involving human participants were reviewed and approved by the ethics board of West China Hospital, Sichuan University (No. 20201045). The patients/participants provided their written informed consent to participate in this study.

## Author Contributions

Conceptualization, D-xL and X-mW; formal analysis, D-xL and D-cF; funding acquisition, PH; investigation, D-xL, YB, KC, R-cW, and PH; methodology, XW and F-cZ; resources, D-cF; supervision, PH.; visualization, XS; writing-original draft, D-xL; writing-review and editing, D-cF and PH. All authors contributed to the article and approved the submitted version.

## Funding

The study was supported by the Pillar Program from Department of Science and Technology of Sichuan Province (2018SZ0219) and the 1.3.5 project for disciplines of excellence, West China Hospital, Sichuan University (ZY2016104).

## Conflict of Interest

The authors declare that the research was conducted in the absence of any commercial or financial relationships that could be construed as a potential conflict of interest.

## Publisher’s Note

All claims expressed in this article are solely those of the authors and do not necessarily represent those of their affiliated organizations, or those of the publisher, the editors and the reviewers. Any product that may be evaluated in this article, or claim that may be made by its manufacturer, is not guaranteed or endorsed by the publisher.

## Abbreviations

PLMR, Preoperative lymphocyte-to-monocyte ratio; ILMP, Lymphocyte-to-monocyte ratio during induction; PILMR, Preoperative dividing by induction lymphocyte -to- monocyte ratio; TURBT, Transurethral resection of bladder tumor; NMIBC, Non-muscle-invasive bladder cancer; BCG, Bacillus Calmette-Guérin; HV, Histological variant; WHO, World Health Organization; SD, Standard Deviation; HR, Hazard ratio; BMI, Body mass index; CI, Confidence level; RFS, Recurrence-free survival; PFS, Progression-free survival.
